# Evidence and consensus on technical aspects of embryo transfer

**DOI:** 10.1093/hropen/hoac038

**Published:** 2022-09-06

**Authors:** Arianna D’Angelo, Costas Panayotidis, Alessandra Alteri, Saria Mcheik, Zdravka Veleva

**Affiliations:** Wales Fertility Institute, Swansea Bay Health Board, University Hospital of Wales, Cardiff University, Cardiff, UK; Attiki Iatriki Advanced Gynaecological Ultrasound and Hysteroscopic Centre Private Practice, Pallini, Athens, Greece; IRCCS San Raffaele Scientific Institute, Milan, Italy; European Society of Human Reproduction and Embryology (ESHRE) Central Office, Strombeek-Bever, Belgium; Helsinki University Central Hospital, Helsinki, Finland

**Keywords:** ART, embryo transfer, good practice, IVF, pregnancy, recommendations, ultrasound

## Abstract

**BACKGROUND:**

Ultrasound-guided embryo transfer (US-GET) is a widely performed procedure, but standards for the best practice are not available.

**OBJECTIVE AND RATIONALE:**

This document aims to provide an overview of technical aspects of US-GET after considering the published data and including the preparation for the embryo transfer (ET) procedure, the actual procedure, the post-procedure care, associated pathologies, complications and risks, quality assurance and practitioners’ performance.

**SEARCH METHODS:**

A literature search for evidence on key aspects of the ET procedure was carried out from database inception to November 2021. Selected papers (n = 359) relevant to the topic were analysed by the authors. The following key points were considered in the papers: whether ultrasound (US) practice standards were explained, to what extent the ET technique was described and whether complications or incidents and how to prevent such events were reported. In the end, 89 papers could be used to support the recommendations in this document, which focused on transabdominal US-GET.

**OUTCOMES:**

The relevant papers found in the literature search were included in the current document and described according to the topic in three main sections: requirements and preparations prior to ET, the ET procedure and training and competence for ET. Recommendations are provided on preparations prior to ET, equipment and materials, ET technique, possible risks and complications, training and competence. Specific aspects of the laboratory procedures are covered, in particular the different loading techniques and their potential impact on the final outcomes. Potential future developments and research priorities regarding the ET technique are also outlined.

**LIMITATIONS, REASONS FOR CAUTION:**

Many topics were not covered in the literature review and some recommendations were based on expert opinions and are not necessarily evidence based.

**WIDER IMPLICATIONS:**

ET is the last procedural step in an ART treatment and is a crucial step towards achieving a pregnancy and live birth. The current paper set out to bring together the recent developments considering all aspects of ET, especially emphasizing US quality imaging. There are still many questions needing answers, and these can be subject of future research.

**STUDY FUNDING/COMPETING INTEREST(S):**

No funding. A.D.A. has received royalties from CRC Press and personal honorarium from Cook, Ferring and Cooper Surgical. The other co-authors have no conflicts of interest to declare that are relevant to the content of this article.

WHAT DOES THIS MEAN FOR PATIENTS?

In this review, we aimed to advise on the best practice for the ultrasound-guided embryo transfer (US-GET) procedure based on the available evidence (both published articles and expert opinion). As the final step in assisted reproduction, expertise and precision in use of this technique is critical for success of the treatment, i.e. achieving a live birth. The authors attempted to establish a standardized protocol for US-GET to improve pregnancy and live birth rates, and to minimize exposure of women to unnecessary or harmful interventions. In addition, an optimal US-GET is needed to avoid multiple treatment cycles, the high expenses of which make IVF inaccessible to many, especially in developing countries. A more effective embryo transfer procedure also helps reduce the physical and psychological burden after failed cycles and would lead to fewer couples giving up before achieving a pregnancy. We have also identified the gaps in research and the need for new trials in order to optimize and standardize the US-GET technique for clinical practice, as some interventions have not been shown to be beneficial for patients or lack sufficient evidence in support of their effectiveness and safety.

## Introduction

Embryo transfer (ET) is the procedure in which one or several embryos are placed into the uterine cavity. It is the final and one of the most critical steps within ART for both patient and doctor.

The effectiveness of the ET procedure is evaluated by the success rate of a commencing pregnancy using three parameters: the positive pregnancy test (urine or blood), the ultrasound (US) verification around 6–7 weeks confirming a gestational sac and/or embryo cardiac activity, and ultimately the live birth rate.

A plethora of published papers on ET technique (Tıras and Cenksoy, 2014; De los Santos *et al.*, 2016; Practice Committee of the American Society for Reproductive Medicine, 2017; Saravelos and Li, 2019) demonstrates that different protocols, different approaches and system set-ups are followed within clinics, some of which are reported to be associated with improved outcomes after ART (Tıras and Cenksoy, 2014; Saravelos and Li, 2019). However, to date, no international consensus on ET standards of practice has been established. One possible explanation is that success of the ET procedure depends on many factors, several of which are difficult to standardize and hence to investigate. Among these are operator experience, difficulty in catheterization, embryo catheter loading technique (air bubbles, culture medium characteristics, volume of fluid), pressure and speed of injection, duration of the ET and US settings.

This article summarizes the recent evidence on ET technique through a review of the literature. It further provides some practical recommendations for practitioners and formulates standards for future ET practice, based on the collected evidence and expert opinion. The recommendations focus on ultrasound-guided embryo transfer (US-GET), in which US guidance is transabdominal.

Transabdominal US guidance is the gold standard procedure performed for ET. Transmyometrial US-GET is performed in extremely rare cases, e.g. a resistant cervix or the presence of other anatomical obstacles (e.g. severe stenosis on the cervical part, repetitive failed mock ET) (Sharif *et al.*, 1996; Groutz *et al.*, 1997). A transvaginal US technique for ET has also been described (Porat *et al.*, 2010; Bodri *et al.*, 2011; Larue *et al.*, 2017). Because of their rare use, transmyometrial US-GET, transvaginal US-GET and clinical touch ET (where the clinician tactilely judges the correct catheter position without technical assistance) were considered to be outside the scope of this paper.

## Methods

**Figure 1.**
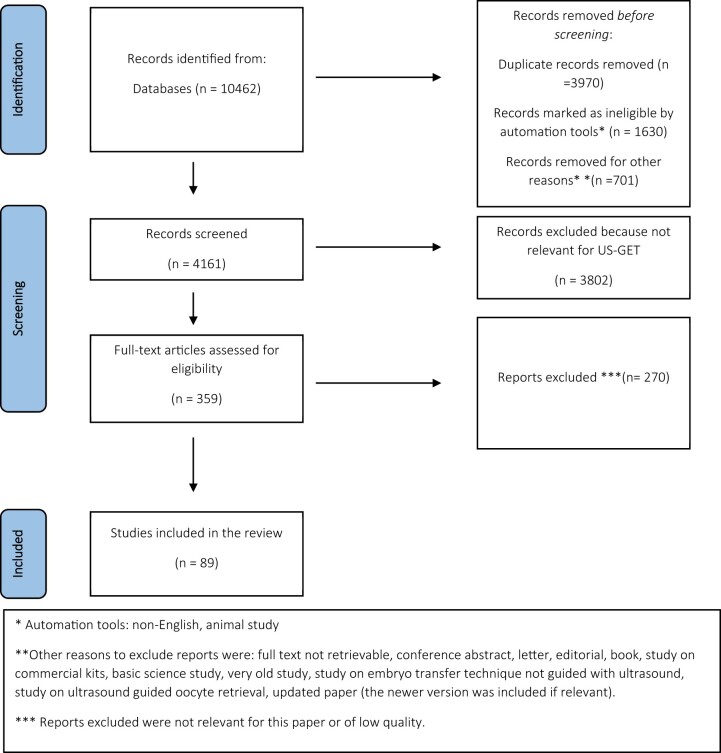
**Flow chart of the selection of papers included in the narrative review of the technical aspects of embryo transfer.** US-GET, ultrasound-guided embryo transfer.

PUBMED, Embase and the Cochrane database were searched from inception to November 2021 for papers on the topic of ET technique. References (titles and abstracts) were screened and considered against the defined inclusion criteria (English language, human studies) and exclusion criteria [publication type (editorial, letter, book, studies on commercial kits, basic science studies), reviews of which a more recent version is available, not on topic of US-GET] (Fig. 1). Only papers focusing on US-GET were selected and papers on other techniques for ET were excluded. For references considered to be relevant, full-text papers were collected, assessed and summarized by the appointed author, depending on the topic (Fig. 1). Further information from guidelines and regulatory papers was added based on the experience and research of the authors.

## Recommendations

**Figure 2.**
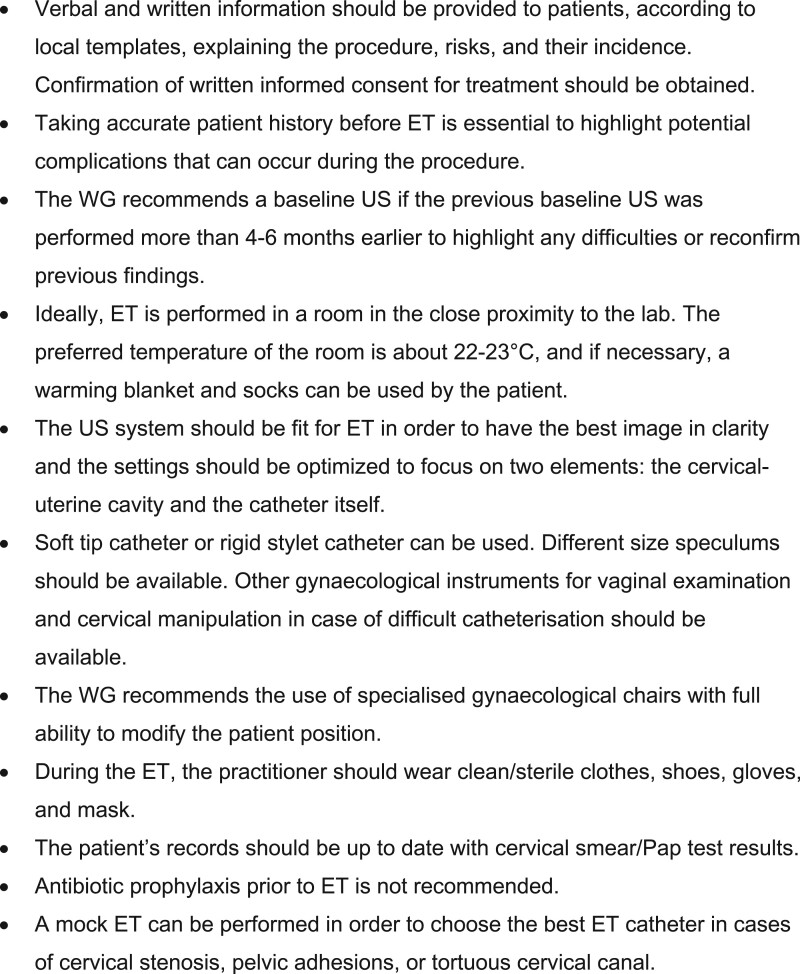
**Recommendations regarding the requirements and preparations prior to embryo transfer.** ET, embryo transfer; US, ultrasound; WG, working group.

**Figure 3.**
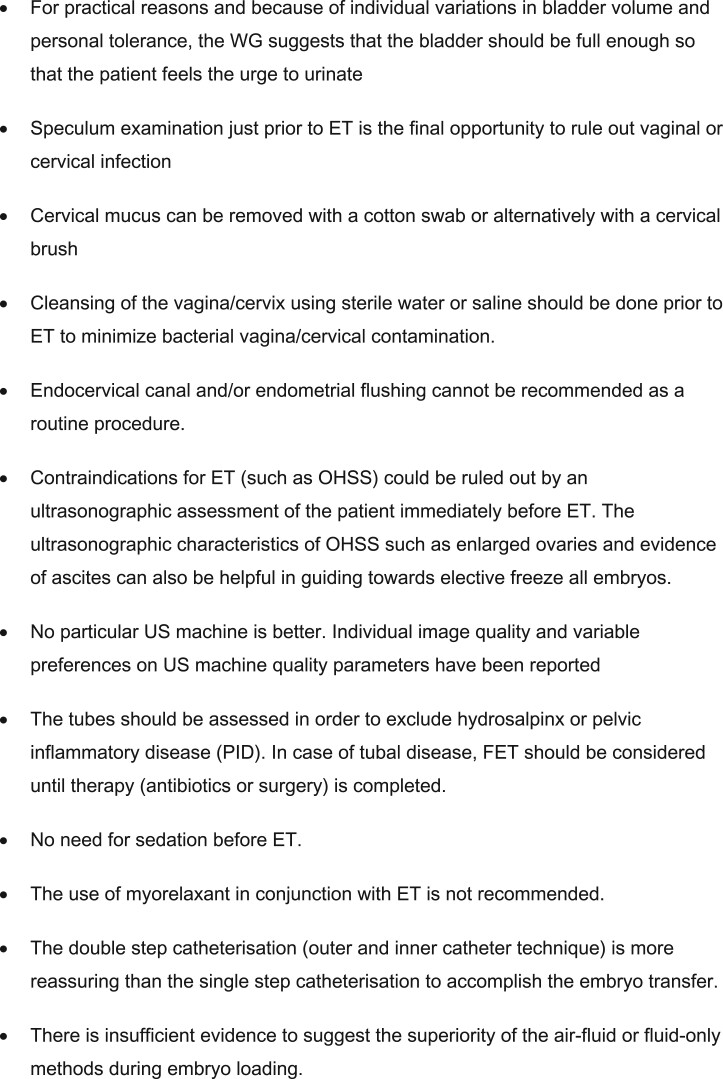

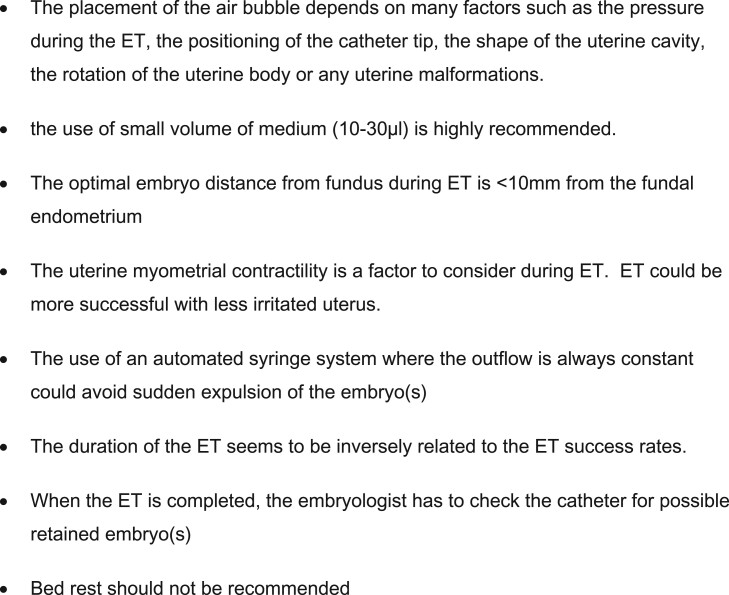
**Recommendations regarding the embryo transfer procedure.** ET, embryo transfer; WG, working group; OHSS, ovarian hyperstimulation syndrome; FET, frozen embryo transfer.

**Figure 4.**
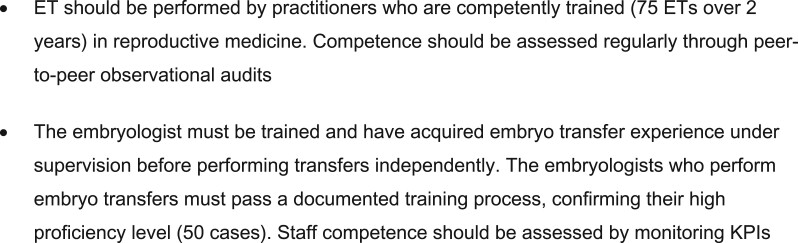
**Recommendations regarding quality assurance and performance.** ET, embryo transfer; KPIs, key performance indicators.

The literature search resulted in 89 papers being included in the current review and described according to topic in three main sections: requirements and preparations prior to ET, the ET procedure and training and competence for ET. A summary of all the recommendations is listed in Figs 2–4.

### Requirements and preparations prior to ET

#### Requirements prior to ET

Preparation for ET follows roughly the same steps as the preparation for oocyte retrieval in terms of infection screening and disinfection requirements (D'Angelo *et al.*, 2019). However, there are several important differences. There is usually no need for sedation prior to ET, while filling the urinary bladder and ruling out ovarian hyperstimulation syndrome (OHSS) are additional steps that are required and are specific to ET.

**Figure 5.**
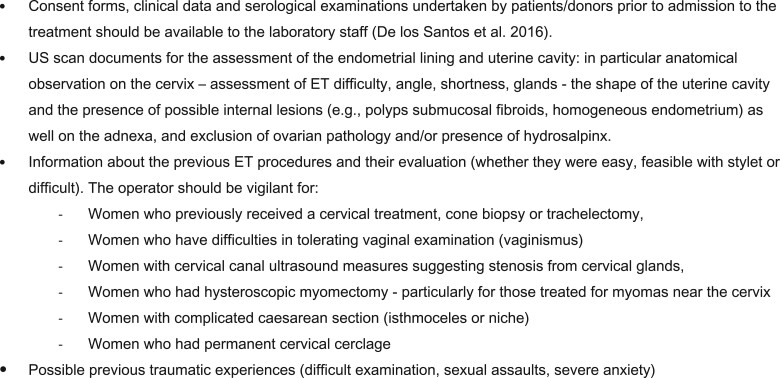
**List of items to be checked before embryo transfer.** ET, embryo transfer.

**Figure 6.**
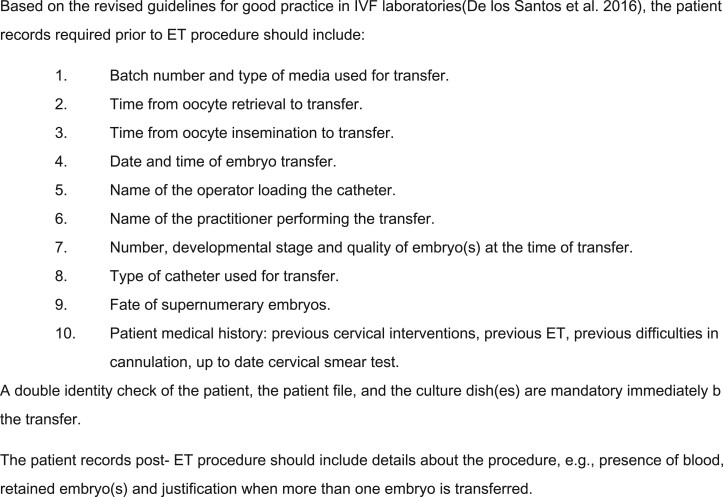
**Patient information required on the patient records.** ET, embryo transfer.

Patients should be provided with preparatory information about the ET procedures (Gameiro *et al.*, 2015). It is also important to review the patient’s file before planning for ET and, in case of a concern, to consider performing a mock ET in advance. Figure 5 summarizes the list of items to be checked prior to ET and Fig. 6 summarizes the information required on the patient’s record before ET is carried out.

#### Preparations prior to ET

##### Pelvic US

A pelvic US evaluation should be performed before starting ART, to decide on the ovarian stimulation protocol and to determine whether there is an anatomical abnormality of the uterus or malposition of the ovaries (Grimbizis *et al.*, 2016). This baseline diagnostic US examination also allows for the detection of recent lesions, such as endometrial abnormalities or ovarian cysts, in a timely manner and is helpful to visualize the uterus and predict potential difficulties during ET. The time frame to perform the US is at the discretion of the clinician. The working group (WG) recommends a baseline US, if the previous baseline US was performed more than 4–6 months earlier to highlight any difficulties or reconfirm previous findings (D'Angelo *et al.*, 2019). This time frame should be shortened in cases of significant conditions (endometriosis, surgery, specific symptoms).

##### Uterine measurement prior to ET

A large, randomized controlled trial (RCT) comparing transabdominal US-GET with a technique based on uterine length measurement before ET found no difference between the two techniques in terms of success, but the ET based on uterine length was better tolerated and easier to perform as a single operator was needed (Revelli *et al.*, 2016).

Similarly, a prospective study found that US-GET does not enhance pregnancy rates compared with ET based on previous uterine length measurement (Lambers *et al.*, 2006). However, there seems to be a relation between uterine length and successful implantation (Chun *et al.*, 2010).

Uterine cavity measurement has been proposed as a strategy to improve ET outcomes, however, studies in the literature failed to show any cut-off value.

##### Doppler study

Variable practices are noted with regards to the Doppler study. It could be of value in cases of adenomyosis and for evaluating the maturity of the endometrium. Three-dimensional (3D)-US and power Doppler angiography can offer a comprehensive assessment of endometrial and sub-endometrial vascularization by using three indices: vascularization index, flow index and vascularization-flow index, which are important factors underlying endometrial receptivity.

Extensive work suggests that Doppler pulsatility index evaluation may be predictive of ET success and that better vascularity gives better intra-myometrial and sub-endometrial blood flow (Cacciatore and Tiitinen, 1996). Chien *et al.* (2002) observed better ET results in patients with the presence of both endometrial and sub-endometrial flow in comparison with patients with sub-endometrial flow only or no detectable endometrial-sub-endometrial flow. In their meta-analysis, Wang *et al.* (2018) concluded that endometrial and sub-endometrial vasculature may be associated with the ET outcome.

##### Uterine 3D-US examination

3D-US and Doppler investigations are considered helpful for the operator to become familiar with the patient’s anatomy, the shape of the uterine cavity, the myometrium, the presence of possible cervical stenosis and to measure the cervical canal (D'Angelo *et al.*, 2019). Some authors have performed 3D-US just before, during and after ET and reported the latter provided them with information about the location of the pregnancy in relation to where the embryo was deposited (Letterie, 2005; Fang *et al.*, 2009). Yet, an RCT comparing 3D versus 2D US-GET has demonstrated that 3D US-GET is an imaging technology that does not improve clinical outcomes compared with 2D US-GET (Saravelos *et al.*, 2016). The ongoing pregnancy rates between 3D- and 2D-US groups were not significantly different (35.4% versus 37.1%, *P* = 0.070, risk ratio 0.96, 95% CI 0.75–1.21). 3D-US prior to ART could be considered as a complementary imaging study benefiting certain individual patients (e.g. in case of suspected or previous difficult ET).

#### Operating room, equipment and consumables

Ideally, ET should be performed in a room in close proximity to the laboratory to minimize exposure of the embryos to temperature drops. If the laboratory is some distance from the ET room, arrangements should be made to maintain temperature and pH while transporting embryos (De los Santos *et al.*, 2016). The preferred temperature of the ET room is 22–23°C (D'Angelo *et al.*, 2019).

All essential items, equipment and supplies required for ET should be available. During the ET, the practitioner should wear clean/sterile clothes, shoes, gloves and a mask in accordance with European standards and local regulations.

There are no studies investigating the association between the US system quality used for ET and ET outcomes. Yet, the US system should have the ability to: adjust the field of view, depth and zoom; adjust the focal zone to the region of interest; adjust the acoustic power, colour and power Doppler capabilities; display the mechanical and thermal indices on screen; display the catheter guide superimposed on the field of view; and to print or save images/cine loops in the system’s hard drive or a central picture archiving and communication system, including image gain adjustment controls (D'Angelo *et al.*, 2019). Settings that can increase the contrast of the different tissues or increase the black for fluid can be used to avoid artefacts.

A soft tip catheter or rigid stylet catheter can be used for the ET. The choice of transfer catheter can be based on the following characteristics: a design that is less traumatic, length, tip diameter, lumen opening at the tip for the embryo(s) passage, cost, echogenicity of the body and tip, and, overall, their consistency and whether they are easy to manipulate. A meta-analysis of two RCTs and two cohort studies showed that the pregnancy rates were improved when soft catheters were used compared to rigid catheters (Practice Committee of the American Society for Reproductive Medicine, 2017).

Gynaecological instruments for vaginal examination and cervical manipulation in case of a difficult catheterization should be available. These include speculum, vorcellum/tenaculum, dilators, sponge holder, cotton swabs, cotton buds for cervical mucus, forceps and a cleaning set including saline/cleaning solution.

A standard medium-size Cusco-type speculum is the most commonly used, but different sizes of speculum should also be available. However, in specific patients (e.g. obese patients, or those with previous vaginal surgeries) other types of speculum can be used, such as Colin’s or Grave’s.

#### Patient and practitioner positioning

Gynaecological positioning of the patient in the semi-lithotomy or lithotomy can facilitate the ET performance. The position of the patient may need to be adapted to patient mobility.

The gynaecological examination chair should provide optimal comfort for the patient and ergonomic positioning for the doctor and assistant. The WG recommends the use of specialized gynaecological chairs with full ability to modify the patient position.

#### Mock ET

A mock ET (or a dummy ET) can be performed in a preceding cycle (Ali *et al.*, 2008), at the time of oocyte retrieval (Mirkin *et al.*, 2003) or immediately before ET (Prapas *et al.*, 1995). It has been shown not to increase the frequency of uterine contractions (Torre *et al.*, 2010). A mock ET can diminish the incidence of difficult transfers by allowing the physician to choose the most suitable catheter. Other potential benefits, such as an accurate measurement of the uterine cavity length, are most beneficial in settings where US is not available at ET (Shamonki *et al.*, 2005). However, most patients who proceed to ET might have already had mapping of the cervical canal, through previous IUIs or sonohysterography. Moreover, US guidance during ET is widely available nowadays. US can be used for moulding the ET catheter to the uterocervical angle (Sallam *et al.*, 2002). It should also be noted that a retroverted uterus will often change position, even to anteversion, thus challenging the effectiveness of a mock ET (Henne and Milki, 2004).

A mock ET seems to be less valuable in case of US-GET and in the presence of a thorough documentation of observations of previous transcervical procedures. Nonetheless, it can be performed in order to choose the best ET catheter in cases of cervical stenosis, pelvic adhesions or tortuous cervical canal.

#### Prevention of infection

The patient’s records should be up to date with cervical smear/PAP test results. A vaginal infection screening (including, if necessary, a vaginal swab for bacteriological examination) should be performed during diagnostic work-up according to local guidelines and regulations.

The evidence from the literature does not support the administration of prophylactic antibiotics in association with ET. A systematic review of the literature in 2012 concluded that prophylaxis with amoxicillin and clavulanic acid did not improve IVF success rates (Kroon *et al.*, 2012). Similarly, a retrospective study of 876 fresh and frozen-thawed transfers, with or without oral doxycycline and methylprednisolone, found no independent effect of antibiotic prophylaxis at ET on the success of treatment (Kaye *et al.*, 2017).

A survey of IVF clinics in the USA reported that 40% of them use antibiotic prophylaxis (Beshar *et al.*, 2021); the authors of this study conducted a retrospective analysis of the transfer of 250 single euploid blastocysts in frozen cycles and showed that doxycycline prophylaxis did not result in higher live birth and ongoing pregnancy rates.

In women with symptoms of infection, it is recommended to perform specific microbiological testing and take appropriate actions (D'Angelo *et al.*, 2019). In general, antibiotic prophylaxis should be used only when supported by evidence, since it can induce resistance and can have negative side effects, including *Clostridium difficile* and fungal infection (Shirlow *et al.*, 2017).

Regarding vaginal screening, the literature search did not provide information on this topic.

#### Associated pathologies and factors affecting success

The successful implantation requires a receptive intrauterine environment for the embryo(s) and the presence of uterine pathologies can negatively affect the success rate of the ET.

Preparation for a difficult ET is required in cases of the following associated pathologies: uterine malformation, fibroids, obesity, endometriosis and post-surgery pelvic inflammatory disease (PID). Ideally, these pathologies are detected in the pre-ET US and either treated or considered in the preparation of a difficult ET.

The use of screening hysteroscopy may reveal intrauterine pathologies that may not be diagnosed by transvaginal US and the use of hysteroscopic surgery to optimize the uterine cavity (e.g. septum resection) may be of value.

On the one hand, a Cochrane systematic review including 11 RCTs showed that there is insufficient data to decide whether routine screening hysteroscopy increases live birth and clinical pregnancy, be it for all women or those with two or more failed IVF attempts (Kamath *et al.*, 2019). A recent RCT assessed the role of office hysteroscopy prior to the first ART cycle and the authors reported that the office hysteroscopy did not improve ART results. Minimal intrauterine anomalies not diagnosed by transvaginal US or hysterosalpingography do not seem to negatively affect ART outcomes (Ben Abid *et al.*, 2021). On the other hand, there is low-quality evidence that operative hysteroscopy increases the pregnancy rate in infertile women with previously diagnosed polyps (Di Spiezio Sardo *et al.*, 2016).

More robust and high-quality RCTs are needed to demonstrate the benefit of diagnostic and/or operative hysteroscopy before ET in the general population. Thus, this intervention could not be recommended based on the existing evidence.

### Patient preparation for ET

Before carrying out the ET, the identity of the patient should be checked and the World Health Organization surgical safety checklist applied (World Allaince for Patient Safety, 2008).

#### A full urinary bladder

The patient should attend the ET procedure with a full bladder. This straightens the angle between the uterine cervix and uterine body (Sundström *et al.*, 1984; Lewin *et al.*, 1997) and facilitates visualization using the transabdominal US scan. A straighter cervical canal and smaller inclination of the uterine body facilitate the effortless insertion of an ET catheter into the correct spot in the uterine cavity (Abou-Setta, 2007). It was suggested in a large study by Lewin *et al.* (1997) that performing ET with a full bladder increases the clinical pregnancy rate. Two smaller RCTs failed to show such an effect (Mitchell *et al.*, 1989; Lorusso *et al.*, 2005). A difference in fluid intake instructions may account for the difference in results: Lewin *et al.* (1997) instructed patients to take 1000 ml of liquids, whereas Mitchell *et al.* (1989) required only 250 ml, and Lorusso *et al.* (2005) required a ‘moderately filled’ bladder.

For practical reasons and because of individual variations in bladder volume and personal tolerance, the WG suggests that the bladder should be full enough so that the patient feels the urge to urinate. If necessary, after the initial assessment, the patient can be instructed to drink an additional one to two cups of water in order to achieve optimal distension of cervico-uterine angle. Excessive distension of the bladder can cause significant discomfort to the patient, and the assistant may not be able to produce the best US images despite the posterior bladder enhancement of the image. However, by optimal positioning of the patient, the anteverted uterus can change its position by force of gravity, even with smaller bladder volumes. Therefore, patient positioning and use of gynaecological couches that feature bottom and back tilting with fully optimizable leg support, can avoid significant technical difficulties in ET and improve the patient experience with the procedure.

In the case of a retroverted uterus, a full bladder makes the uterine-cervical angle more pronounced and therefore more difficult to catheterize.

#### Speculum examination

Speculum examination just prior to ET is the final opportunity to rule out vaginal or cervical infection. The practitioner should also evaluate the external appearance of the cervix in order to rule out potential signs of difficult ET such as cervical polyps or cervical ectropion/inflammation.

##### Removing mucus from the cervical canal

Removing mucus from the cervical canal can facilitate the insertion of an ET catheter into the uterine cavity and it can potentially avoid a clogged catheter tip, or relocation of mucus within the uterine cavity, which may affect implantation. On the other hand, removing cervical mucus might stimulate uterine contractility or cervical bleeding, which can have a negative effect on the ET outcome.

Cervical mucus can be removed with a cotton swab or a cervical brush, although the latter is considered to be slightly more traumatic with a higher risk of provoking uterine contractions. Careful catheter aspiration of mucus is another option. In an early trial, Mansour *et al.* (1994) injected methylene blue dye into the uterine cavity in a mock ET and concluded that expulsion of the dye was significantly reduced after the removal of cervical mucus. One RCT in which cervical mucus was removed with sterile cotton swabs (Moini *et al.*, 2011) and a prospective cohort study of catheter aspiration (Eskandar *et al.*, 2007) demonstrated that removing mucus improved clinical outcomes. However, a meta-analysis of eight RCTs (including the previously cited RCTs) involving 1715 women reported very little evidence of an overall benefit of cervical mucus removal before ET (Craciunas *et al.*, 2014). A similar conclusion was reported by a Cochrane meta-analysis, even if the methods of mucus removal and studies included are questionable (Derks *et al.*, 2009).

#### Disinfection

Cleansing of the vagina/cervix should be carried out prior to ET to minimize bacterial vaginal/cervical contamination. Currently, most practitioners achieve this by using sterile water or saline. Cleaning prior to ET should be performed delicately in order to avoid bleeding. This is important not only for the success of ET catheterization but also for diminishing the subsequent stress of the patient if she detects spotting after ET.

#### Flushing the endocervical canal and endometrial cavity prior to ET

A Cochrane meta-analysis including studies on the effect of flushing the endocervical canal or the endometrial cavity on pregnancy rates found no evidence of any substantial benefit (Derks *et al.*, 2009). Owing to the lack of benefit, endocervical canal and/or endometrial flushing cannot be recommended as a routine procedure.

#### Pelvic US immediately prior to ET

The aim of the US assessment of the patient immediately before ET is to rule out contraindications for ET. Among contraindications for ET, OHSS is the most common and potentially life-threatening. While the ultimate decision on cancelling ET because of OHSS relies also on laboratory findings and subjective symptoms, the US characteristics of OHSS, such as enlarged ovaries and evidence of ascites, can also help guide towards elective freeze-all of embryos (D'Angelo and Amso, 2002).

US prior to ET additionally aims to confirm a beneficial uterine environment and endometrium, i.e. an endometrial thickness of preferably >7 mm (Kasius *et al.*, 2014). Although optimal results from ART can be achieved in patients with regular uterine cavities with no deformities (e.g. septa, fibroids or polyps), a small study on patients with uterine polyps up to 15 mm in length, some of which were treated through hysteroscopic resection, reported that the presence of small polyps was not associated with poorer pregnancy and implantation rates (Isikoglu *et al.*, 2006).

Occasionally, fluid in the uterine cavity at the time of ET can be observed in patients with hydrosalpinx whose tubes communicate freely with the uterine cavity (i.e. those who have not undergone salpingectomy or tubal obliteration) (Melo *et al.*, 2020).

The presence of intrauterine fluid prior to ET seems to be an unfavourable prognostic factor; the tubes should be assessed in order to exclude hydrosalpinx or PID. Elective freeze-all of the embryos should be considered until therapy of tubal disease (antibiotics or surgery) is completed (Melo *et al.*, 2020).

#### Pain relief and uterine myorelaxant

It is believed that patient feedback during ET is important in ensuring an atraumatic procedure with minimal uterine contractions and minimal disruption to the endometrium.

A trial evaluated the use of phenazopyridine, a bladder analgesic, for reducing discomfort during ET (Frishman *et al.*, 2007). A single dose of the medicine, administered 1 h prior to ET, failed to reduce discomfort, as measured with a visual analogue pain scale. In the American Society for Reproductive Medicine guidelines on ET, acupuncture, analgesics, massage, general anaesthesia and traditional Chinese medicine were listed as having no beneficial effect on pregnancy (Practice Committee of the American Society for Reproductive Medicine, 2017). While not necessarily required for ET, variable sedation techniques have been reported, including the use of sedative drugs, such as Propofol, Rapifen, Fentanyl and Diazepam. Verbal analgesia by the seditionist/assistant is another important anxiolysis form (D'Angelo *et al.*, 2019).

### The ET procedure

There are two potential ET practices: single step and double step ET.

The single-step option involves the use of a prepared soft catheter. These catheters have a very smooth and flexible inner part and a second external tube to protect the inner part as well to give more stability during the insertion. With this concept, internal cervical/uterine trauma is avoided and the embryo(s) can be passed through the cervical canal. There is a risk, however, that difficulties are encountered in passing through the cervical canal, or even that it is impossible to pass the soft catheter.

In the double-step option, a rigid double catheter is used and passed through the cervical canal up to the top end of the cervix. The second step is to remove the inner part and replace it with the softer catheter containing the embryo(s). Although the double-step option may increase the risk of cervical/internal trauma or increase patient discomfort, it is more reassuring for the operator to successfully pass and complete the ET.

#### Catheter loading

The embryo loading technique represents a critical aspect of the procedure and might affect ART outcomes. The choice of the syringe, type of catheter, type and volume of transfer medium, presence of air bubble, catheter loading speed and embryo(s) placement in the catheter may be variables involved in the success of the procedure.

**Figure 7.**
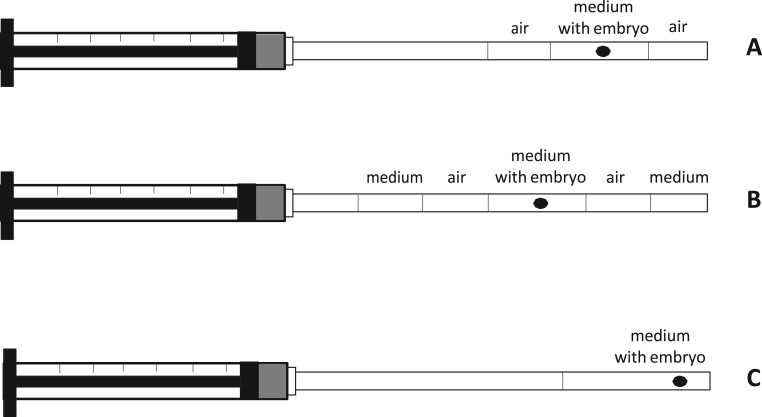
**Catheter loading methods used in human embryo transfer.** (**A** and **B**) Air–fluid method: the transfer media containing the embryo(s) separated by air spaces on both sides. (**C**) fluid-only method: the embryo(s) is placed in a complete column of fluid without any air brackets or bubbles.

Two main catheter loading methods have been described: the air–fluid method (air–embryo–air or medium–air–embryo–air–medium) (Fig. 7A and B, respectively) and the fluid-only method (Fig. 7C).

In the air–fluid method, the loading of the syringe–catheter complex with the transferred volume consists of the transfer media (which contains the embryo(s)) separated by air spaces on both sides. In the fluid-only method, the embryo(s) is placed in a complete column of fluid, without any air brackets or bubbles. A systematic review and meta-analysis of two prospective randomized trials concluded that there was insufficient evidence to suggest the superiority of the air–fluid or fluid-only methods during embryo loading (Abou-Setta *et al.*, 2007).

Moreover, the effect of medium volume and the presence of air bubbles on clinical outcomes appear to be controversial. Generally, the use of a small volume of medium (10–30 µl) is highly recommended. Indeed, a large volume of transfer medium (>60 µl) may increase the chance of dislocation of the transferred embryo(s) from the uterus into the cervix or Fallopian tubes, predisposing to ectopic pregnancy. Likewise, a very small medium volume (<10 µl) along with air bubbles seemed to have a negative effect on implantation and pregnancy rates (Ebner *et al.*, 2001). A comparison between 40–50 and 15–20 µl showed that a higher volume is associated with increased implantation and pregnancy rates (Montag *et al.*, 2002). However, some studies found no difference in terms of clinical outcomes between low (15–25 µl) and high (35–45 µl) transfer volume (Omidi *et al.*, 2015; Sigalos *et al.*, 2018).

Of note is that loading the catheter directly from the culture micro drop under the oil versus loading from the transfer dish without an oil layer leads to similar pregnancy rates (Halvaei *et al.*, 2013).

Air bubbles might help with US visualization of the ET catheter and proper placement of the embryo(s) (Schoolcraft, 2016). The use of air bubbles in the catheter might also protect the embryos from the cervical mucus and accidental discharge before entering the endometrial cavity (Tiras *et al.*, 2012). The embryo is within the surface of this bubble and follows the flow of the liquid used as propulsion, usually to the top part of the uterine cavity (floating).

#### ET technique and procedure

Once the resolution of the cervical image on the US is optimized, the practitioner attempts to pass the flexible tip of the catheter directly through the cervical canal under US guidance. If an angled uterine body in relation to the cervix is detected, this can be corrected by manipulating the speculum holder ascending or descending the initial portion of the cervix situated between the speculum valves. The possibility to adjust the pelvic part of the gynaecological couch aiming to descend the bottom of the patient and the back simultaneously can be very helpful and makes the uterine body lower (due to gravity) and aligned with the cervical canal.

The practitioner should try to pass the catheter as smoothly as possible in an axial trajectory without bending it or irritating the patient’s cervix and/or endometrium. Bending, repeated attempts to catheterize or difficulties to progress within the uterine cavity can result in irritation of the myometrium, creating micro-contractions (Fanchin *et al.*, 1998). Two studies concluded that during the ET those who had better IVF outcome had a less irritated uterus (Sammali *et al.*, 2018; Blank *et al.*, 2020). However, the use of atosiban, an oxytocin receptor antagonist, to reduce the uterine contraction during ET is unlikely to improve the clinical pregnancy rate or the live birth rate in the general IVF patient population (Buddhabunyakan *et al.*, 2021). The clinical pregnancy rate in older women (>35 years old) in the atosiban group was twice that of the placebo group, but the result was not statistically significant (Buddhabunyakan *et al.*, 2021).

3D-US and the four-dimensional-US with abdominal probes can demonstrate the positioning of the embryo(s) or show more realistically the catheter in relation to the uterine cavity. Reports showed 80% accuracy of the embryo(s) positioning with 3D-US imaging just after ET with a subsequent scan confirming an intrauterine pregnancy (Baba *et al.*, 2000).

**Figure 8.**
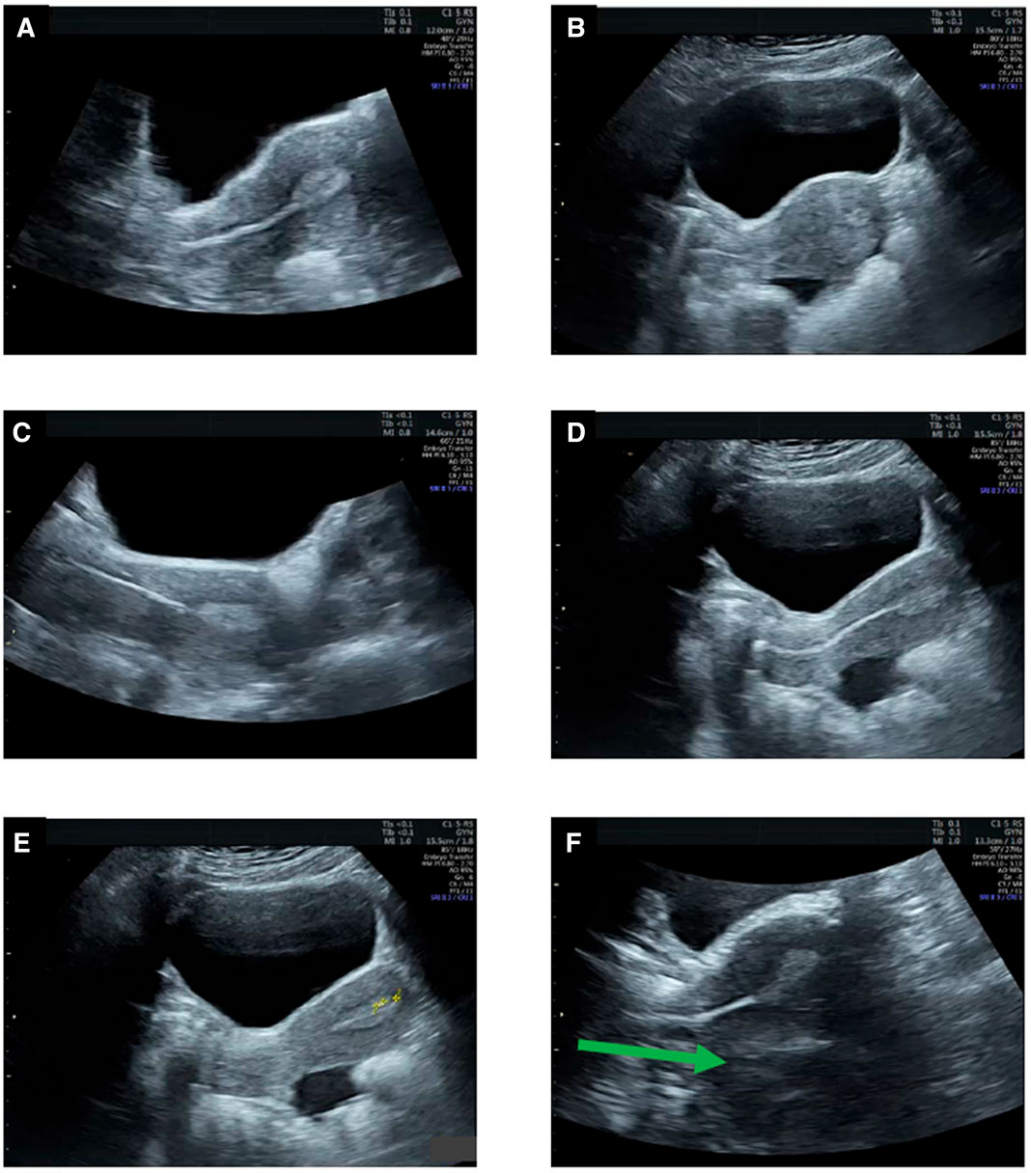
**Ultrasound images showing various factors related to a transabdominal ultrasound-guided embryo transfer.** (**A**) Zoom window focused on the uterine cavity. (**B**) Deep field view of embryo transfer (ET) during catheterization. (**C**) Retroverted uterus ET. (**D**) ET catheter approaching the fundal part of the endometrium. (**E**) Measurement of distances between tip of catheter and fundal endometrium and between embryo bubble and endometrium. (**F**) Artefacts during ET: example of mirror image artefact (uterus is anteverted but there is a false image showing a retroverted uterus).

To improve the US image, the assistant can apply gentle pressure on the patient’s abdomen, although a similar effect can be reached by adjusting the US settings on more recent US systems (Fig. 8).

Extensive work on identifying the optimal place to release the embryo has taken place because the movement of the final position of the embryo is unlikely to be a factor predicting the success of ET (Allahbadia *et al.*, 2008; Ozcan *et al.*, 2016). The middle upper area gives better results in terms of implantation and pregnancy rates (Oliveira *et al.*, 2004). Cavagna *et al.* (2006) similarly suggested avoiding ET in the lower regions of the uterine cavity, as this may result in higher miscarriage rates.

Higher pregnancy rates were obtained when the position of the air bubble from the fundal endometrial surface was <10 mm. When the inner catheter tip is placed 1.5–2 cm from the fundal endometrium, a best performance is expected (Cenksoy *et al.*, 2014). In a more recent retrospective study, it was shown that the probability of pregnancy, clinical pregnancy and ongoing pregnancy decreases as the distance from the fundus (DFF) to the air bubble (SD: 10.27 ± 3.0 mm) increases (Bayram *et al.*, 2021). When all variables remained constant, an increase of 1 mm of DFF changed the odds of pregnancy by 0.882; of clinical pregnancy by 0.891 and of ongoing pregnancy by 0.925.

There are no studies to evaluate what is the best way to withdraw the catheter, i.e. as inner and outer together, or separately.

##### Pressure in the piston

Some authors have proposed an automated syringe system where the outflow is always constant (Caanen *et al.*, 2016) with the potential benefit to avoid sudden expulsion of the embryo(s).

The injection pressure during the ET is related to the catheter length and therefore if the catheter end is in the proximity of the fundal end, one should use the lowest pressure possible in order to complete the ET with a gentle injection and maintain the embryo(s) as close to the upper fundal area as possible. In cases of larger uterine cavities, the ET catheter end may not achieve ideal positioning towards the fundal end. Chun *et al.* (2010) reported a possible higher miscarriage rate when the embryo bubble did not reach the highest point.

##### Duration of the ET procedure

The duration of the ET procedure is the time taken for: the embryologist to prepare the loaded catheter; catheter transport to operator hands; the catheterization (one step or two steps), passing through the cervix and moving the catheter tip to the targeted point within the endometrial cavity; the injection; and catheter withdrawal. The duration of the ET procedure has been shown to have a significant influence on pregnancy success rates, with a duration of the transfer of more than 120 s having a negative effect (Matorras *et al.*, 2004).

A plausible explanation for the association between duration of the procedure and ET outcomes could be the time during which embryo(s) is(are) outside the incubator as well as the difficulty of ET. The duration of the ET seems to be inversely related to the ET success rates (Abdelmassih *et al.*, 2007). Cetin *et al.* (2010) observed that the highest ET success rates were noted when the time from when the embryo(s) was loaded on the catheter to the time when the embryo(s) was released was <44 s. They showed a 35% success rate for young women (<35 years old). However, for the difficult ETs, the time was detrimental for the older women (>35 years old) and if the ET time is more than 60 s, the difference between the two groups was significant, with 30% success for women <35 years old and 13% success for women >35 years old.

#### End of procedure and post-procedure care

When the ET is completed, the embryologist has to check the catheter for possible retained embryo(s). This is a crucial quality control procedure. The embryo should be reinjected immediately; a retrospective analysis of data from 12 studies showed that the implantation rate, the clinical pregnancy rate and the pregnancy loss rates were not decreased for patients undergoing immediate re-transfer after embryo retention (Practice Committee of the American Society for Reproductive Medicine, 2017).

A Cochrane review (Abou-Setta *et al.*, 2014) and two systematic reviews (Craciunas and Tsampras, 2016; Cozzolino *et al.*, 2019) looked at bed rest after ET and found that immediate mobilization after ET does not influence success rates. Therefore, bed rest should not be recommended.

### Complications and troubleshooting

#### Complications and risks associated with the ET procedure

Complications during ET (e.g. the presence of cervical trauma/bleeding, retained embryo in the catheter, rare expulsion of the embryo from the cervix and short-term post-ET infection) are very rare. Long-term complications in ART are failure to achieve pregnancy, ectopic pregnancy, miscarriage and multiple pregnancies.

Although the majority of ETs are straightforward, some degree of difficulty can be encountered, even if there is no consensus on what qualifies as a difficult ET. Generally, ETs have been defined as difficult when they cause discomfort to the patient or when there is the presence of blood at the end of the catheter. There may be some anatomical difficulty to access the uterine cavity and, in this case, the ET requires the use of specific tools such as catheters with sheaths and rigid mandrels (stylet). In addition, the embryo may be retained in the transfer catheter, which is usually detected by the embryologist and requires a repeated transfer.

The effect of difficult transfers on clinical outcomes is debated. While some studies reported no harmful effect of difficult transfers, others reported detrimental effects on clinical outcomes (Arora and Mishra, 2018).

#### Troubleshooting during ET

In cases of cervical catheterization difficulties, where the catheter does not pass the cervical canal, only partially passes or is bent, a more forced catheterization can be attempted using a tenaculum to stabilize the cervix (traction), a thicker stylet catheter or external cervical dilators. In case of a complete cervical catheterization failure, it is advised to proceed to freeze all of the embryos and hysteroscopic assessment.

An important point to consider in troubleshooting is when to abandon the procedure and when to try again, for example, with another catheter (e.g. change to a rigid one) or resort to gentle dilatation of the cervix. There are very limited data informing such a decision. Tur-Kaspa *et al.* (1998) showed that in case of a difficult ET, uterine manipulation or cervical dilatation or repeated attempts at the time of the ET could be performed without adversely affecting the pregnancy outcomes. Until further conclusive data are available, it can be recommended that the practitioner decides in a case-by-case approach on the most appropriate course of action.

### Quality assurance and performance

#### Training and competence

##### Clinicians

Two RCTs confirmed that US-GET performed by either midwives or experienced nurses does not impact negatively on the outcomes (Bjuresten *et al.*, 2003; Rinaldi *et al.*, 2014).

The assistant’s experience does not seem to impact outcomes following US-GET (Harris *et al.*, 2009). Thus, assistance during ET for someone without formal US training is a reasonable option.

ET should be performed by practitioners who are competently trained in reproductive medicine. In some countries, fertility specialists or nurses can be trained to perform ET procedures, but there are currently no generally accepted minimal requirements for training. For safety reasons, and wherever feasible, a simulator could be the initial part of structured training for novices who want to perform this procedure, enabling them to acquire basic skills and reach a predefined level of performance in a safe and controlled environment before applying the procedure to patients (Soave *et al.*, 2019).

The number of procedures to be completed for training (within 2 years) is 75 according to the recently published paper based on the Maribor Consensus (Vlaisavljevic *et al.*, 2021).

In addition to training, competence in a certain procedure should be maintained. Criteria for assessing proficiency/competency on the technical aspects of ET have not been described, but suggested criteria are pregnancy rates, number of ETs performed relative to the size of the clinic and ectopic pregnancy rates.

Competence should be assessed regularly through peer-to-peer observational audits, the frequency of which should be decided within the team.

##### Embryologists

The embryologist must be trained and have acquired ET experience under supervision before performing transfers independently. The embryologists who perform ET must pass a documented training process, confirming a high proficiency level. Each laboratory has its own training programme that includes: reading and understanding the standard operating procedures; observing the ET procedures performed by qualified and experienced embryologists; loading of discarding material without any loss; and performing a minimum of 50 ET procedures under supervision. After reaching pregnancy rates within two standard deviations of the average pregnancy rate in the laboratory/clinic, the trainee can be authorized to perform the ET procedure without supervision (Montag and Morbeck, 2017). A good training programme includes training on how to deal with difficulties and problems such as embryo return, re-loading of embryos and options for difficult transfers.

Maintaining embryologists’ competence is critical as well. To demonstrate each embryologist’s expertise, a certain procedure number should be recorded in a logbook (Alpha Scientists in Reproductive Medicine, 2015). For example, ESHRE requires the completion of 50 cases in 3 years for the ET procedure in order to evaluate the competency of a Clinical Embryologist (Kovačič *et al.*, 2020).

Staff competence should be assessed by monitoring key performance indicators (ESHRE Special Interest Group of Embryology and Alpha Scientists in Reproductive Medicine, 2017) (Table I). The maintenance of achieved competence should be monitored annually, even for senior embryologists and if necessary, re-training is recommended.

**Table I:** Staff training and competence in performing embryo transfer.

	Ovarian stimulation and trigger	Oocyte retrieval	Embryo transfer
Training		Number of procedures to complete	
	100 cycles^*^	75^*^	75^*^
Competence		Monitor PIs to check competence and skills Take appropriate action when there is a gap between actual and expected performance	

* The numbers are those proposed by the ESHRE clinic PI working group.

Table adapted from Vlaisavljevic *et al.* (2021).

PI, performance indicators.

### Future developments

Transvaginal US-GET has some benefits such as no need for a full bladder avoiding bladder discomfort, the practitioner is doing both transvaginal sonography (TVS) and ET with no need for assistance, and the TVS has a far better image. However, the process to catheterize prior to the transvaginal US-GET takes more time for preparation than a standard ET under the transabdominal US-GET and it is unclear what could be done in case of a difficult ET (Bodri *et al.*, 2011). Bodri *et al.* (2011) did not show superior success rates between transvaginal US-GET and standard transabdominal US-GET.

Cozzolino *et al.* (2018) concluded that in three recent RCTs the quality of evidence supporting the equivalence of the transvaginal versus transabdominal approach in clinical pregnancy or live birth rates is low and they identified the need for larger RCTs.

Future research should focus on factors and methods that could increase the ET success rate. An association between the US system quality used for ET and the outcomes has not yet been investigated; whether optimizing the image quality can help practitioners avoid cervical catheterization difficulties, manipulate the ET catheter more gently and complete the ET procedure in an atraumatic and precise way is not known.

More evidence-based knowledge is needed regarding the catheter loading techniques to compare the air-fluid or fluid-only methods during embryo loading and also regarding the best way to withdraw the catheter (as inner and outer together, or separately).

## Conclusion

ET is the last procedural step in ART and is crucial for achieving a pregnancy and live birth. The current paper set out to bring together recent developments concerning all aspects of ET, especially emphasizing US quality imaging. There are still many questions needing answers, and these can be the subject of future research.

What is clear is that the performance of ET is not researched in-depth and objective data that are based on US criteria are not routinely recorded or checked. The sequence of what steps are needed to perform ET is similar in most fertility clinics around the world.

Although ETs are performed frequently, clear standards and quality criteria to improve their effectiveness are needed.

The authors’ opinion is that US quality combined with a gentle tactile technique can make a difference in pregnancy rate improvement at ET.

## Data Availability

This article conducts a literature review of existing research records, and no new data were generated or analysed in support of this manuscript.
